# Compound Comminuted Fracture of the Spinal Vertebrae, Extending from the Second Lumbar to the Base of the Sacrum, Exposing Cauda Equina—Operation and Recovery

**Published:** 1904-07

**Authors:** E. M. Albers

**Affiliations:** Pinos, Zac., Mexico


### TEXAS MEDICAL JOURNAL.

ESTABLISHED JULY, 1885.

PUBLISHED MONTHLY.—SUBSCRIPTION $1.00 A YEAR.

VOL. XX. AUSTIN, JULY, 1904. No. 1.

### Original Contributions.

For Texas Medical Journal.

### Compound Comminuted Fracture of the Spinal Vertebrae, Extending From the Second Lumbar to the Base of the Sacrum, Exposing Cauda Equina—Operation and Recovery.

BY E. M. ALBERS, M. D., PINOS, ZAC., MEXICO.

On the 16th of October, 1901, while surgeon of the Harbor Works Company of Manzanillo, Mexico, Juan Montana, a native laborer, age 30, was brought to my office for surgical treatment, having just received an injury by a rock falling on his back.

Examination revealed the following conditions: An injury of some seven inches in length, over, and involving, the spinal column, extending from the second lumbar vertebræ to the base of the sacrum, freely exposing the cauda equina.

Under strict asepsis, without an anesthetic (no pain was present) I removed all detached fragments, elevated all depressed and excised all ragged pieces of bone. The patient bore the operation well. The wound was dressed aseptically and all after dressings were aseptic. An ice-pack was placed over site of wound and continuously applied for the four succeeding days, when it was temporarily discontinued for twenty-four hours.

Up to this time the patient rested well, was free from pain, temperature and pulse normal.

During the first four days following the injury, retention of urine existed, necessitating catherterization; on the fifth day the bladder voluntarily resumed its function; a cystitis supervened and persisted for several weeks, finally disappearing without special treatment. Paralysis of the bowel existed during the first seven days, and on the eighth day the bowel voluntarily resumed its function.

Fifteen hours after the discontinuance of the ice-pack the following clinical history was present. The patient, for the first time, complained of great weakness, continuance indicating marked depression:

21st: 7 a. m.—temperature, 101 degrees; pulse, 110; respiration, 20.

21st: 4 p. m.—temperature, 102¾ degrees; pulse, 125; respiration, 26.

(Reapplied the ice-pack at 5 p. m.)

21st: 9 p. m.—temperature, 101½ degrees; pulse, 105; respiration 18.

22d: 7 a. m.—temperature, 99 degrees; pulse, 65; respiration, 14.

22d: 4 p. m.—temperature, 99½ degrees; pulse, 60; respiration, 15.

On and after the 23d the pulse and temperature remained normal. The wound healed without suppuration.

Six weeks after the injury sensory sensation below seat of injury unimpaired; motor somewhat impaired; patella reflexes exaggerated, the walk of the patient showing some incoordination of the muscles of locomotion, seemingly indicating an incipient locomotor ataxia. One year after injury the patient was fully restored to health.

The proximity of the spinal cord and its anatomical relation with the cauda equina rendered it necessary to prevent inflammation, which would no doubt extend by continuity to the cord or its membranes.

I am under the impression that the temporary withdrawal of the ice-pack on the fifth day was the cause of the partial paralysis of the muscles of locomotion; but so slight was the inflammatory induration that secondary absorption restored the function of the nerves to the paralyzed muscles.

The object of this article is to direct special attention to the value of the ice-pack in the treatment of wounds in a hot, sultry climate; in these localities my experience has been that wounds, accidental or operative, have a tendency to inflame and suppurate, even in the presence of strict asepsis.

